# Supplementary material for: Ultrasound-guided medial branch of the superior laryngeal nerve block to reduce peri-operative opioids dosage and accelerate patient recovery
Source: PLoS One. 2023 Dec 11;18(12):e0295127. doi: 10.1371/journal.pone.0295127 (PMC10712872; doi:10.1371/journal.pone.0295127)
Supplement: S1 Protocol — (DOCX) [file pone.0295127.s003.docx]

**Introduction**

The concept of enhanced recovery after surgery (ERAS) refers to the application of various proven effective methods before, during and after surgery to reduce surgical stress and complications and accelerate the postoperative recovery of patients [1]. The implementation of ERAS is based on evidence-based medicine with the main purpose of accelerating rehabilitation. At the same time, it is necessary to take into account the specific conditions of patients' underlying diseases, types of surgery, and perioperative complications, and further clinical studies are needed to demonstrate the safety, feasibility and necessity of ERAS related pathways. ERAS preoperative intervention mainly includes: preoperative education, preoperative visit and evaluation, optimization of preoperative fasting (drinking carbohydrate drinks), optimization of intestinal preparation, appropriate antibacterial prophylaxis, prevention of deep vein thrombosis (DVT), and avoidance of long-term antianxiety drugs. Intraoperative intervention mainly includes: local and epidural anesthesia, minimizing the use of drainage tube and nasogastric tube, avoiding excessive volume, maintaining normal body temperature, and preventing postoperative nausea and vomiting. Postoperative interventions mainly include: optimizing diet plan, controlling blood glucose, early removal of catheter and restoration of enteral nutrition, minimizing the use of opioid analgesics, and getting out of bed as soon as possible [2]. In recent years, in the promotion of ERAS, the reduction and standardization of the use of opioids has been paid more and more attention. In terms of pain management, opioid analgesics are the main drug in traditional programs, but they are prone to postoperative adverse reactions such as nausea and vomiting, urine retention, intestinal obstruction and respiratory depression, resulting in prolonged hospital stay and recovery period. The ERAS protocol recommends avoiding the use of long-acting opioids, adopting regional and local anesthesia techniques, and alternatives to opioids such as non-steroidal drugs, anti-inflammatory drugs, acetaminophen, etc. [3]. Therefore, it is currently believed that reducing the use of opioids in the perioperative period is conducive to rapid recovery of patients after surgery.

The postoperative pain of patients undergoing laparoscopic abdominal surgery mainly comes from the surgical incision. Therefore, blocking nerve conduction from this source can greatly reduce postoperative pain. Transversus abdominis plane block (Tap block) is a regional anesthesia technique in which a local anesthetic is injected into the fascial plane between the internal oblique and transverse abdominis muscles to block nerve conduction in the somatic innervation area from T6 to L1. Thus, the pain sensation in the skin, muscles and parietal peritoneum of the anterior abdomen is weakened to achieve analgesic effect [4]. TAP block and the use of opioid substitutes in laparoscopic surgery can have sufficient analgesic effect on surgical incision. However, when anesthesia is induced, sufficient opioids are still needed to inhibit the intense stress response caused by tracheal intubation.

The superior laryngeal nerve is a branch of the vagus nerve that runs along the lateral wall of the pharynx and is divided into internal and external branches at the greater horn of the hyoid bone. The internal branch and the artery of the same name accompanied the thyrohyoid periosteum into the larynx, distributed in the laryngeal mucosa above the glottic cleft, the epiglottis and the base of the tongue. The external branch accompanied by the superior thyroid artery ran forward and down, 0.5~1.0cm from the superior thyroid, leaving the curved artery to the medial side, and issuing the muscular branch to innervate the cricothyroid muscle and the subpharyngeal retractor muscle. Superior laryngeal nerve block can inhibit laryngeal pharyngeal sensation and inhibit the reflex response of glottis and some subglottic trachea mucosa to foreign body stimulation [5]. It has been reported that the application of superior laryngeal nerve block in awake tracheal intubation can effectively inhibit the cough and cardiovascular response of patients during intubation, and improve the comfort of patients [6]. In addition, it can also be used for transesophageal echocardiography and endoscopy, such as laryngoscopy, bronchoscopy, esophagoscopy, and gastroscopy, and other surgeries requiring larynx and esophagus [5]. Therefore, we speculate that the use of internal branch block of superior laryngeal nerve to inhibit the stress response caused by tracheal intubation can further reduce the perioperative opioid consumption of patients undergoing laparoscopic surgery and accelerate the postoperative rehabilitation of patients.

References

[1].Yeung Sung Ching,Irwin Michael G,Cheung Chi Wai,Environmental Enrichment in Postoperative Pain and Surgical Care: Potential Synergism With the Enhanced Recovery After Surgery Pathway.[J] .Ann. Surg., 2020, undefined: undefined.

[2].Visioni Anthony,Shah Rupen,Gabriel Emmanuel et al. Enhanced Recovery After Surgery for Noncolorectal Surgery?: A Systematic Review and Meta-analysis of Major Abdominal Surgery.[J] .Ann. Surg., 2018, 267: 57-65.

[3].Tan Mingjuan,Law Lawrence Siu-Chun,Gan Tong Joo,Optimizing pain management to facilitate Enhanced Recovery After Surgery pathways.[J] .Can J Anaesth, 2015, 62: 203-18.

[4].McDonnell John G,O'Donnell Brian D,Farrell Thomas et al. Transversus abdominis plane block: a cadaveric and radiological evaluation.[J] .Reg Anesth Pain Med, 2007, 32: 399-404.

[5].Fu Y H,Min X,Long P,[The anatomic characteristics and surgical treatment of congenital pyriform sinus fistula].[J] .Lin Chung Er Bi Yan Hou Tou Jing Wai Ke Za Zhi, 2018, 32: 984-987.

[6].Monsó A,Riudeubàs J,Palanques F et al. A new application for superior laryngeal nerve block: treatment or prevention of laryngospasm and stridor.[J] .Reg Anesth Pain Med, 1999, 24: 186-7.

**Research objectives**

The application of internal branch block of superior laryngeal nerve to inhibit the stress response caused by tracheal intubation can further reduce the perioperative and intraoperative opioid consumption in patients undergoing laparoscopic surgery and provide new technical support for accelerating postoperative rehabilitation of patients undergoing laparoscopic surgery.

**Research program**

1. Sample size estimation: The sample size of this study was estimated on the basis that the total amount of sufentanil during operation in pre-trial group B (10 patients) was 0.235± 0.047ug /kg, and the total amount of sufentanil during operation in conventional treatment group D (10 patients randomly selected) was 0.38 ± 0.132ug /kg, unilateral α=0.05, 1-β=0.9. f (0.05,0.1) = 10.5. Therefore, a sample size of 17.4 was required per group, taking into account a possible loss of follow-up rate of 15%, and 20 patients were recruited per group.

Sample selection: A total of 20 patients, aged 18-65 years, 145-185cm in height, 45-80kg in weight and ASA grade I to III, were selected for elective laparoscopic surgery and endotracheal general anesthesia in our hospital.

Inclusion criteria: (1) ASA grade I ~ III; (2) Age 18-65 years old; (3) Height 145-185cm; (4) Weight 45-80kg; (5) Education level above primary school; (6) Sign informed consent.

Exclusion criteria: (1) Participating in other clinical trials within 4 weeks before surgery; (2) severe heart, lung, liver, kidney and other organ dysfunction; (3) Have a history of neurological and mental illness, and a history of long-term use of sedatives; (4) have severe aphasia, visual and hearing impairment, serious movement impairment, can not cooperate with the examination; (5) Pregnant or lactating women; (6) Patients with motion sickness and allergy to ropivacaine; (7) Intraoperative blood loss greater than 500ml; (8) The surgical incision was enlarged during the operation.

2. 80 patients with laparoscopic surgery were selected, and the ASA grades were Ⅰ~Ⅲ. Patients were divided into four groups by simple randomization. Group A: bilateral superior laryngeal nerve block (2% lidocaine 3ml) + abdominal transversal fascia block group, sufentanil induction dose 0.4ug/kg; Group B: bilateral superior laryngeal nerve block (2% lidocaine 3ml) + abdominal transverse fascia block group, the induced dose of sufentanil was 0.2ug/kg; Group C: bilateral superior laryngeal nerve block (2% lidocaine 3ml) + abdominal transversal fascia block group, sufentanil induced 0ug/kg; Group D was the conventional control group: bilateral superior laryngeal nerve block (3ml normal saline) + abdominal transversal fascia block group, the induced dose of sufentanil was 0.4ug/kg.

3. All patients underwent endotracheal aspiration after entering the room, which was consistent with general anesthesia. Specific protocols were as follows: The patient was given midazolam 0.04mg/kg, propofol 0.2mg/kg, superior laryngeal nerve block, induction of atracurium cis-benzoate 0.2mg/kg (other induction drugs vary according to different groups), positive mask pressure ventilation for 3 minutes, and endotractal intubation under visual laryngoscope. Sevoflurane 1%, propofol 0.05mg/(kg.min), remifentanil 0.06ug/(kg.min) were used for maintenance. Concurrent transversal fascia block.

4. Implementation of blind method: The trial designer randomly enrolled the patients, numbered them, and equipped them with treatment drugs (sufentanil diluted into the same volume with normal saline); By the test operator, the patients underwent superior laryngeal nerve block and transverse abdominal fascia block, and were induced and managed under general anesthesia. The test data is collected and sorted by the data collector.

5. Record the patient's general data, including heart rate, blood pressure and oxygen saturation at the time of entry, before induction, immediately after intubation, 3min after intubation and 5min after intubation. Anesthesia time, operation time, remifentanil and sufentanil dosage were recorded. The blood pressure before extubation, 1min after extubation and 5min after extubation were recorded in the recovery room, and the residence time of patients in the recovery room was recorded. The frequency of postoperative sore throat, nausea and vomiting were recorded. The time of urinary retention and intestinal exhaust were recorded, and the numeric rating scales (NRS) of resting and exercise pain at 3h, 6h, 12h, 24h and 48h after surgery were recorded, as well as whether analgesics were used in the ward and the type and dosage. Record other special circumstances. 48 h after follow-up, the satisfaction of patients was expressed on a scale of 1 to 10, with 10 being perfect.

6. Handling of special conditions during the operation:

(1) Treatment of intraoperative hemodynamic changes: anesthesiologists with more than 3 years of clinical experience shall give appropriate treatment according to specific conditions and make good records.

(2) Treatment of postoperative incision pain, nausea and vomiting: the doctors in the recovery room and ward shall give corresponding treatment and make records. (3) Local anesthetic poisoning treatment: immediately stop the injection of local anesthetic drugs, and protect patients to avoid accidental injury. Take oxygen and perform assisted or controlled breathing. Maintain hemodynamic stability. In the case of cardiac arrest (which has a very low probability of occurrence), 1.5ml/kg of 20% fat emulsion was injected intravenically, followed by 0.25ml/(kg.min) of intravenous infusion, cardiopulmonary resuscitation, and electrical defibrillation if necessary.

**Expected results**

Tracheal intubation is a clinical operation often needed by anesthesiologists. There are literature reports that the use of superior laryngeal nerve block can reduce the stress response of patients during awake tracheal intubation, reduce the secretion of catecholamines in the blood of patients and reduce hemodynamic fluctuations. The purpose of this study is to investigate whether the application of internal branch block of superior laryngeal nerve can inhibit the stress response caused by tracheal intubation, further reduce the perioperative opioid consumption of laparoscopic surgery patients, and accelerate postoperative rehabilitation. However, the final effect is subject to the experimental data analysis results, without personal subjective will.

**Feasibility analysis**

1. The project is based on a solid theoretical foundation: By referring to a large number of domestic and foreign literatures, we have grasped the latest research progress on superior laryngeal nerve block, which provides a strong theoretical basis for this subject.

2. Department of Anesthesiology, Affiliated Hospital of Guilin Medical College has advanced ultrasound and other anesthesia monitoring instruments, which can provide hardware support for the experiment.

3. The Affiliated Hospital of Guilin Medical College has enough laparoscopic operations, which can provide abundant medical records for this experiment.

4. The teachers of the research group have rich clinical ability, scientific research strength and teaching experience, and can give full help and guidance on the subject.

**The innovation of this research topic**

The application of internal branch block of superior laryngeal nerve combined with transverse abdominal fascia block in patients with laparoscopic ERAS was studied by combining the two regional block techniques to provide new technical support for accelerating postoperative rehabilitation of patients with laparoscopic surgery.
