# Supplementary material for: Ultrasound-guided medial branch of the superior laryngeal nerve block to reduce peri-operative opioids dosage and accelerate patient recovery
Source: PLoS One. 2023 Dec 11;18(12):e0295127. doi: 10.1371/journal.pone.0295127 (PMC10712872; doi:10.1371/journal.pone.0295127)
Supplement: Attachment — Submitted filename: Response to Reviewers.docx [file pone.0295127.s004.docx]

Dear reviewers

We would like to thank you for your careful reading, helpful comments, and constructive suggestions, which has significantly improved the presentation of our manuscript.

We have carefully considered all comments from the reviewers and revised our manuscript accordingly. The manuscript has also been double-checked, and the typos and grammar errors we found have been corrected. All abbreviations,particularly in the abstract and introduction have been defined. In the following section,we summarize our responses to each comment from the reviewers. We believe that our responses have well addressed all concerns from the reviewers.

In our revisions, we paid specific attention to the comments

1) “The introduction needs more context, including an overview of perioperative pain management importance, postoperative pain prevalence, and the significance of reducing opioid usage, with supporting statistics or evidence for multimodal analgesia.” For this comment, we describe the importance of perioperative pain management in the first paragraph of the introduction according to the existing literature.We have reviewed the corresponding literature and explained the prevalence of postoperative pain at the beginning of the second paragraph of the introduction. At the end of third paragraph of the introduction, we state the significance of reducing opioid usage.
2) “Clarify the sample size justification, whether based on primary outcomes.”For this comment,our answer is “Based on our pilot study,the heart rate change before and after intubation in group B was 11.7±2.3 beats/minutes, and the change in group A was 19±6.6 beats/minutes. Thus, the sample size was calculated with a power of 0.8 and alpha error of 0.05. At least 17.4 subjects per group were needed. Assuming a 10% dropout rate and to increase the credibility of this study, 80 patients were recruited (20 subjects for each group).”The above content has been added to the article.
3) “Expand the discussion to interpret results in the context of existing literature, discuss implications for perioperative pain management, study limitations, and future research directions”. For this comment, We added the content " Role of multimodal analgesia in ERAS" in the first paragraph of our discussion.We also further interpret the results in the context of existing literature.We have reviewed the shortcomings of this article and made corresponding modifications.We point out that whether special populations (such as patients with airway difficulties, severe cardiovascular diseases, and elderly patients, etc.) can benefit from this protocol and how the medial branch block of the superior laryngeal nerve can shorten the first postoperative intestinal exhaust time and postoperative hospitalization time are worthy further study.
4) “Provide a concise summary of key findings and their implications”. For this comment, we summarized a paragraph at the end of the main text.

All the changes are marked out in the paper ”Revised Manuscript with Track Changes”

Thank you and best regards.

Yours sincerely,

2023.8.20

Corresponding author:

Name: Xu Zhang

E-mail: 450047830@qq.com
