# Supplementary material for: Ultrasound-guided medial branch of the superior laryngeal nerve block to reduce peri-operative opioids dosage and accelerate patient recovery
Source: PLoS One. 2023 Dec 11;18(12):e0295127. doi: 10.1371/journal.pone.0295127 (PMC10712872; doi:10.1371/journal.pone.0295127)
Supplement: Attachment — Submitted filename: Response to Reviewers-1.docx [file pone.0295127.s005.docx]

Dear academic editor and reviewers

We would like to thank you for your careful reading, helpful comments, and constructive suggestions, which has significantly improved the presentation of our manuscript.

We have carefully considered all comments from the reviewers and revised our manuscript accordingly. The manuscript has also been double-checked, and the typos and grammar errors we found have been corrected. All abbreviations,particularly in the abstract and introduction have been defined. In the following section,we summarize our responses to each comment from the reviewers. We believe that our responses have well addressed all concerns from the reviewers.

In our revisions, we paid specific attention to the comments

1) “To make the study more clear, in the introduction, separate the primary and secondary goals” and“Clearly define primary and secondary outcomes, and mention any safety outcomes”

For these comment, We rewrote the last paragraph of the introduction as follows：“Our primary goal was to assess whether the medial branch block of the superior laryngeal nerve can reduce the stress response of patients undergoing general anesthesia during intubation. Our secondary goals were to assess whether the combination of other nerve blocks that can alleviate the incision pain can improve the perioperative pain of patients, thereby further reducing the dosage of opioids and speed up patient's postoperative recovery.”

2) “In the "materials and methods" section, move information about intervention doses and the control group to the randomization methods and rename it as "randomization." ” and“clarify if blinding and allocation concealment were used”

For this comment,We have made corresponding modifications according to the suggestions, and wrote a section named “Randomization and blinding”

3) “In Figure 1 (CONSORT), move it to the results section and add the numbers in each "allocated to group x." ”.

For this comment, We added the numbers in each "allocated to group x" in Figure 1 (CONSORT). However, if we move Figure 1 (CONSORT) to the results section, it will become Figure 2. Through reading related articles published in PLOS ONE, we found that there are also articles that put Figure 1(CONSORT) in Materials and methods section. So, we have retained Figure 1 (CONSORT) in this section.

4) “Specify your statistical comparisons in advance, and address how missing data will be handled.” and “Revise the sample size section to ensure it's clear whether Groups A and B from the pilot study are similar to this main study, rounding up the number to 18 if necessary”

For this comment, We have made corresponding modifications according to the suggestions. Previously, due to a writing error, we wrote Group B instead of Group C. The largest difference was expected to be between groups A and C which are similar to this main study. The missing data were treated using the mean imputation method.

5) “avoid performing formal tests at baseline since differences observed would be due to chance, so omit p-values”

For this comment, We have made corresponding modifications according to the suggestions.

All the changes are marked out in the paper ”Revised Manuscript with Track Changes”

Thank you and best regards.

Yours sincerely,

2023.11.2

Corresponding author:

Name: Xu Zhang

E-mail: 450047830@qq.com
